# Ma Xing Shi Gan Decoction Attenuates PM2.5 Induced Lung Injury via Inhibiting HMGB1/TLR4/NFκB Signal Pathway in Rat

**DOI:** 10.3389/fphar.2019.01361

**Published:** 2019-11-14

**Authors:** Yu-xiang Fei, Bo Zhao, Qi-yang Yin, Yan-ying Qiu, Guang-hui Ren, Bo-wen Wang, Ye-fang Wang, Wei-rong Fang, Yun-man Li

**Affiliations:** ^1^State Key Laboratory of Natural Medicines, School of Basic Medical Sciences and Clinical Pharmacy, China Pharmaceutical University, Nanjing, China; ^2^Department of Pediatrics, Nanjing Integrated Traditional Chinese and Western Medicine Hospital, Nanjing, China

**Keywords:** Ma Xing Shi Gan Decoction, PM2.5, lung injury, macrophage, inflammatory reaction, HMGB1/TLR4/NFκB pathway

## Abstract

Ma Xing Shi Gan Decoction (MXD), a classical traditional Chinese medicine prescription, is widely used for the treatment of upper respiratory tract infection. However, the effect of MXD against particulate matters with diameter of less than 2.5 μm (PM2.5) induced lung injury remains to be elucidated. In this study, rats were stimulated with PM2.5 to induce lung injury. MXD was given orally once daily for five days. Lung tissues were harvested to assess pathological changes and edema. Myeloperoxidase (MPO) activity and malonaldehyde (MDA) content in lung were determined to evaluate the degree of injury. To assess the barrier disruption, the bronchoalveolar lavage fluid (BALF) was collected to determine the total protein content and count the number of neutrophils and macrophages. For evaluating the activation of macrophage in lung tissue, CD68 was detected using immunohistochemistry (IHC). The levels of inflammatory factors including tumor necrosis factor-alpha (TNF-α), interleukin-1beta (IL-1β), and interleukin-6 (IL-6) in BALF and serum were measured. *In vitro*, a PM2.5-activated RAW 264.7 macrophages inflammatory model was introduced. To evaluate the protective effect of MXD-medicated serum, the cell viability and the release of inflammatory factors were measured. The effects of MXD on the High mobility group box-1/Toll-like receptor 4/Nuclear factor-kappa B (HMGB1/TLR4/NFκB) pathway in lung tissue and RAW 264.7 cells were assessed by Western blot. For further confirming the protective effect of MXD was mediated by inhibiting the HMGB1/TLR4/NFκB pathway, RAW 264.7 cells were incubated with MXD-medicated serum alone or MXD-medicated serum plus recombinant HMGB1 (rHMGB1). MXD significantly ameliorated the lung injury in rats, as evidenced by decreases in the pathological score, lung edema, MPO activity, MDA content, CD68 positive macrophages number, disruption of alveolar capillary barrier and the levels of inflammatory factors. *In vitro*, MXD-medicated serum increased cell viability and inhibited the release of inflammatory cytokines. Furthermore, MXD treatment was found to inhibit HMGB1/TLR4/NFκB signal pathway both *in vivo* and *in vitro*. Moreover, the protection of MXD could be reversed by rHMGB1 in RAW 264.7. Taken together, these results suggest MXD protects rats from PM2.5 induced acute lung injury, possibly through the modulation of HMGB1/TLR4/NFκB pathway and inflammatory responses.

## Introduction

Exposure to respirable urban air particles poses a serious threat to human health across the world (Englert, 2004; Brook et al., 2010; Schneider et al., 2010; Zheng et al., 2015; McGuinn et al., 2019). The toxic effects of particulate matters (PMs) are mainly related to their particle size, and particulate matters with diameter of less than 2.5 µm (PM2.5) can penetrate deeper into the respiratory tract and about 50% of them are retained in the lung parenchyma, causing respiratory infection, cardiovascular disease, and systemic inflammation (Clifford et al., 2018; Nhung et al., 2018; Pope et al., 2018).

Alveolar macrophage, the main phagocyte in lung tissue protects lung tissue against microorganism or particles via phagocytosis (Lukacs et al., 1995). However, when high particulate burdens are deposited and accumulated in the lung, alveolar macrophages will be over-activated and release excessive amounts of inflammatory factors which plays a key factor during the occurrence and development of lung injury (Asimakopoulos et al., 1999; Jacobson et al., 2005; Kallapur and Jobe, 2006; Herold et al., 2011).

Ma Xing Shi Gan Decoction (MXD), a traditional Chinese medicine formula, which was recorded in Treatise on Exogenous Febrile Disease in Eastern Han dynasty has many kinds of pharmacological action including but not limited to anti-asthmatic effect, antiviral effect, and antibacterial effect (Lu et al., 2012; Wang et al., 2012). Currently, MXD is widely used in clinic for treatment of acute upper respiratory tract infection in China (Liao et al., 2017). However, it remains unknown whether MXD has protective effects against PM2.5 induced acute lung injury.

The present study was designed to investigate the protective effect of MXD on PM2.5 induced acute lung injury and to explore the underlying mechanism.

## Materials And Methods

### Reagents

All crude drugs were provided by the pharmacy of Nanjing Integrated Traditional Chinese and Western Medicine Hospital. *Ephedrae herba* (ma huang) collected from the province of Inner Mongolia, China, was purchased from Sanyue Traditional Chinese Medicine Co., Ltd (Nantong China). *Semen armeniacae amarum* (ku xing ren) collected from the province of Hebei, China, was purchased from Jiangsu Huahong Pharmaceutical Technology Co., Ltd (Danyang, China). *Glycyrrhizae radix preparata* (gan cao; licorice) collected from the province of Gansu, China, was purchased from Hangzhou Zhende Traditional Chinese Medicine Co., Ltd (Hangzhou, China). *Gypsum fibrosum* (shi gao; calcium sulfate) collected from the province of Shandong, China, was purchased from Jiangsu Huahong Pharmaceutical Technology Co., Ltd (Danyang, China). The quality of crude drugs is consistent with rule of related part of Chinese Pharmacopoeia (2015 edition). Botanical identifications were authenticated by an associate professor Yong Wang (Nanjing Integrated Traditional Chinese and Western Medicine Hospital, China). Voucher specimens of *Ephedrae herba* (voucher number: No.EH181201), *Semen armeniacae amarum* (voucher number: No.SAA190410), *Glycyrrhizae radix preparata* (voucher number: No.GRP181202), and *Gypsum fibrosum* (voucher number: No.GF181202) have been deposited in the Herbarium of China Pharmaceutical University and registered under the voucher specimen numbers. Standards of Ephedrine hydrochloride (CAS No. 50-98-6, purity 100.0%) and pseudoephedrine hydrochloride (CAS No. 345-78-8, purity 99.8%) were purchased from National Institutes for Food and Drug Control. Standards of Liquiritin (CAS No. 551-15-5, purity ≥98%), Glycyrrhizic acid ammonium salt (CAS No. 53956-04-0, purity ≥98%) and Amygdalin (CAS No. 29883-15-6, purity ≥98%) were purchased from Solarbio Science & Technology Co., Ltd., Beijing, China. PM2.5 was provided by Nanjing Municipal Environmental Monitoring Centre. Rat interleukin-1beta (IL-1β), Interleukin-6 (IL-6) and tumor necrosis factor-alpha (TNF-α) enzyme linked immunosorbent assay (ELISA) kits were obtained from Shanghai MLBIO Biotechnology Co., Ltd., Shanghai, China. The myeloperoxidase (MPO) and malondialdehyde (MDA) assay kits were obtained from Nanjing Jiancheng Bioengineering Institute Nanjing, China. Antibodies to High mobility group box 1 (HMGB1), Toll-like receptor 4 (TLR4), Myeloid differentiation factor 88 (MyD88), and phosphorylated p65 (p-p65) were all purchased from Wanleibio Co., Ltd., Shenyang, China. Antibody to β-actin was purchased from Abways Biotechnology Co., Ltd., Shanghai, China. Antibody to CD68 was purchased from Servicebio Co., Ltd., Wuhan, China. Recombinant HMGB1 (rHMGB1) was purchased from Beyotime Institute of Biotechnology, Shanghai, China. All other reagents were of analytical grade and commercially available.

### Preparation of PM2.5

PM2.5 collecting filters were kindly provided by Jiangsu Environmental Monitoring Center. The atmospheric particulates were collected from 2018.1 to 2018.5 in Nanjing, China. After extraction, a total of 6.76 g PM2.5 was collected. Then the PM2.5 was fully dispersed by ultrasonic washer (40 min, 3 times). The liquid was filtered by 8-fold gauze, and the supernatant was discarded after centrifugation. 2.25 g PM2.5 was obtained by drying. After an ultraviolet sterilization for 40 min, the concentration of PM2.5 suspension was adjusted to 10 mg/ml with deionized water. Before use, the suspension was fully suspended.

### Animals and Experimental Design

Male Sprague-Dawley (SD) rats of SPF level (180–200 g) were purchased from Qinglongshan Animal Farm of Nanjing, China. Animals were housed under a normal 12/12 h light/dark schedule and housed at 24±2 °C with relative humidity (55 ± 5%). Standard chow and water were supplied *ad libitum*.

Rats were divided into six groups: control group, intervention group (MXD, 16.4 g/kg), PM2.5 group (model group), and PM2.5+MXD (4.1, 8.2, and 16.4 g/kg) groups. The administration dosage was calculated according to the content of crude herbs. The middle dose (8.2 g/kg) was 6.3 times of the clinical dosage in human, which equivalent dose calculation is based on body surface area (Nair and Jacob, 2016). One hour after the establishment of acute lung injury, drugs were administrated orally (1 ml/100 g body weight) once daily for 5 consecutive days. Correspondingly, rats in the control group and the model group were treated with normal saline.

All procedures for animal care and use were in accordance with the National Institute of Health (NIH) guidelines for the Care and Use of Laboratory Animals, and approved by the Institutional Animal Care and Use Committee of China Pharmaceutical University [license number: SYXK (Su) 2016-0011]. The numbers of animals for different experimental groups were listed in **Supplementary Table 1** as follows:

### Preparation of MXD

*Ephedrae herba* (ma huang, 4 g) were added into 1 L of double distilled water and decocted for 60 min. After skimming the scum, *Semen armeniacae amarum* (ku xing ren, 12 g), *Glycyrrhizae radix preparata* (gan cao, 8 g), and *Gypsum fibrosum* (shi gao, 24 g) were added and extracted by refluxing with water for another 40 min. Then the crude extract was filtered and the concentration of extract was enriched to 2 g/ml by evaporating (calculated according to the content of crude drugs).

### Quantitative Analysis of MXD By UPLC-MS (Ultra-Performance Liquid Chromatography-Tandem Mass Spectrometry)

Preparation of test solution: MXD was diluted 10,000 times using 50% (v/v) methanol in water. The test solution was obtained after filtration.

Preparation of reference standard solution: The reference standard samples (ephedrine hydrochloride, pseudoephedrine hydrochloride, liquiritin, glycyrrhizic acid ammonium salt, and amygdalin) were weighed accurately and dissolved in 50% (v/v) methanol in water at a concentration of 1 mg/ml. After diluted and pooled, the reference standard solution containing ephedrine hydrochloride (500 ng/ml), pseudoephedrine hydrochloride (500 ng/ml), liquiritin (500 ng/ml), glycyrrhizic acid ammonium salt (500 ng/ml), and amygdalin (500 ng/ml) was obtained.

The preliminary analysis was performed using an Acquity UPLC CSH C18 (2.1×100 mm, 1.7 µm) column in both positive and negative electrospray ionization mode. For positive electrospray ionization mode, mobile phase solvent A was consisted of aqueous phase of ammonium acetate at 5 mmol/L, and mobile phase solvent B was pure methanol. For negative electrospray ionization mode, mobile phase solvent A was ultrapure water, and mobile phase solvent B was methanol. The injection volume was 1 µl. The common elution condition is shown in **Supplementary Table 2**.

The five major components of MXD (ephedrine hydrochloride, pseudoephedrine hydrochloride, liquiritin, glycyrrhizic acid ammonium salt, and amygdalin) were quantified through a UPLC (Acquity UPLC system, Waters, USA)-tandem MS (Xevo TQ-XS mass spectrometer, Waters).

For the analysis of ephedrine and pseudoephedrine in MXD, The UPLC separation was performed at 40 °C using a SHIMADZU VP-ODS (2.0×150 mm) column. Mobile phase solvent A was 0.1% (v/v) formic acid in water, and mobile phase solvent B was acetonitrile. The injection volume was 1 µl. The elution conditions are shown in **Supplementary Table 3**. Positive ionization electrospray MS was employed for the quantification, and the optimal instrument conditions were as follows: cone (V): 30, collision (eV): 12. The precursor-product ion combination of m/z 166.00→148.00 was common quantifier ion for ephedrine and pseudoephedrine. The external standard method is employed for quantitative analysis.

For the analysis of liquiritin and glycyrrhizic acid in MXD, the UPLC separation was performed at 50 °C using an Acquity UPLC HSS T3 (2.1×30 mm, 1.8 µm) column. Mobile phase solvent A was consisted of aqueous phase of ammonium acetate in concentration 5 mmol/L, and mobile phase solvent B was pure methanol. The injection volume was 1 µl. The elution conditions are shown in **Supplementary Table 4**. Positive ionization electrospray MS was employed for the quantification, and the optimal instrument conditions were as follows: cone (V): 42, collision (eV): 8. The precursor–product ion combinations of m/z 419.00→257.00 and 823.41→453.37 were quantifier ion for liquiritin and glycyrrhizic acid. The external standard method is used for quantitative analysis.

For the analysis of amygdalin in MXD, The UPLC separation was performed at 50 °C using an Acquity UPLC HSS T3 (2.1×30 mm, 1.8 µm) column. Mobile phase solvent A was 0.1% (v/v) formic acid in water, and mobile phase solvent B was acetonitrile. The injection volume was 1 µl. The elution conditions are shown in **Supplementary Table 5**. Negative ionization electrospray MS was employed for the quantification, and the optimal instrument conditions were as follows: cone (V): 30, collision (eV): 12. The precursor–product ion combination of m/z 502.00→456.00 was quantifier ion for amygdalin. The external standard method is employed for quantitative analysis.

### Establishment Of Acute Injury

After the grouping, rats were maintained anesthetized with 3% isoflurane in O_2_ for 0.5 h by a respirator mask and fixed in the supine position at a 45-degree angle to horizontal plane. The model of PM2.5 induced acute lung injury was established as previously described with slight modification (Liu et al., 2018). Briefly, a number 16 trocar was inserted into the rat’s trachea, after which the PM2.5 suspension was injected into the trachea through the trocar at a volume of 0.1 ml/100 g body weight. The rats were gently rolled from side to side for 30 s to distribute suspension equably in both lungs. This operation was done at the first, third, fifth, seventh, and ninth day. One hour after the last instillation of PM2.5 suspension, MXD was administered as described in *Animals and Experimental Design* section.

### Histological Analysis

Rats were sacrificed 2 h after the last administration, the left lungs were fixed in 4% formaldehyde (pH = 7.4) overnight and then embedded in paraffin and sliced into 5 µm-thick sections. The histopathological abnormalities of lung tissue were observed under a light microscope after staining with H&E. The pathological changes of the lungs were scored as previously described (Liu et al., 2014; Bao et al., 2015; Lv et al., 2015). The score was graded according to the sum of the score for degree of damage such as the thickening of alveolar walls, the number of infiltration cells and hemorrhage. Each histological characteristic was scored 0 to 5.

### Assessment of Lung Tissue Edema

Two hours after the last administration, rats were sacrificed and lungs were excised and placed on pre-weighed aluminum foil papers. After obtaining wet weight of lungs by weighing, lungs were dried at 90 °C for 48 h in an oven to obtain the dry weight. The water content of the lungs was calculated according to the formula below:

Lung water content (%)=(wet weight−dry weight)/wet weight×100%

### Pulmonary MPO Activity and MDA Content Assay

Five percent homogenate was obtained by homogenizing 0.1 g of right lung tissues in normal saline. The rest right lungs were stored at −80 °C for the following Western blot. MPO which reflects activation of neutrophils and macrophages in lungs was determined using a test kit (Nanjing Jiancheng Bioengineering Institute) according to the instructions (Grattendick et al., 2002; Zhang et al., 2011; Tang et al., 2014). MDA content in lung tissues were detected by an assay kit (Nanjing Jiancheng Bioengineering Institute).

### IHC Assay

Immumohistochemical assay was performed to study the effects of MXD on the expression of CD68, a valuable marker of monocyte/macrophages in the histochemical analysis of inflamed tissues. After the sections (5 µm in thickness) were deparaffinized with xylene and rehydrated with graded ethanol, antigen retrieval was performed in an autoclave (10 min, 121 °C), followed by incubation in 3% H_2_O_2_ at room temperature for 30 min to block endogenous peroxidase activity. After washed with phosphatic buffer solution (PBS), the sections were incubated with goat serum for 30 min at 37 °C, and subsequently incubated with the primary antibody against CD68 (1: 500) for 15 h at 4 °C. Then, the sections were washed with PBS and stained with peroxidase-labeled secondary antibody at 37 °C for 1 h. Slides were then processed using 3, 3-diaminobenzidine chromogenic solution and counter stained with hematoxylin. The pictures were observed using a light microscope (Olympus, Japan, BX53).

### Cell Counting and Protein Concentration in Bronchoalveolar Lavage Fluid (BALF)

Rats were anesthetized 2 h after the last administration. Three milliliters of blood were collected from the abdominal aorta, the serum was then separated at 3,000 g for 15 min and stored at −80 °C for further ELISA tests. The right hilum of the lung was clamped and sutured (to exclude from the lavage) with a 2-0 silk suture thread, after which the right lungs were dissected and stored at −80 °C for MPO assay, MDA assay and Western blot. The tracheae were then cannulated, and the left lungs were lavaged three times with ice-cold PBS (0.5 ml each time) for BALF collection. The mean volume retrieved was 80% of the amount instilled (1.2 ml). The BALF samples were centrifuged at 1,000 g for 5 min. The supernatant was collected and the protein level was measured by a bicinchoninic acid (BCA) assay kit (Jiancheng Bioengineering Institute, Nanjing, China). The cell pellet was resuspended in 100 µl PBS and the number of total cells was counted using a hemocytometer (Qiujing, Shanghai, China). Then cells were centrifuged onto slides and stained with Wright-Giemsa (Wanleibio, Shenyang, China, WLA043a) for 8 min. Differential cell counts were quantified using light microscope by counting a total of 200 cells/slide at 40× magnification. Number of each cell type was calculated as the percentage of cell type multiplied by the total number of cells in the BALF.

### Cytokine Assays in BALF And Serum

The concentrations of TNF-α, IL-6, and IL-1β in the BALF supernatant and serum were measured by ELISA kits (Shanghai MLBIO Biotechnology Co., Ltd., Shanghai, China) according to the instructions. Briefly, specific monoclonal antibody was coated on a 96-well plate. Standards and samples were added to the wells. Next, a biotinylated detection antibody was added, followed by avidin-horseradish peroxidase. 3,3′,5,5′-tetramethylbenzidine (TMB) substrate was subsequently added, producing a blue color. Finally, the reaction system color was changed from blue to yellow by adding the stop solution. Then the absorbance value was measured at 450 nm using a microplate reader (Infinite M200 Pro, Tecan).

### Cell Culture

RAW 264.7 cells, a macrophage cell line which has been widely used as an in vitro inflammatory model, was kindly provided by Prof. Zhong-lin Yang (China Pharmaceutical University), were cultured at 37 °C in a humidified atmosphere of 5% CO_2_/95% air and maintained in DMEM (Kaiji, Nanjing, China) with 10% heat inactivated fetal bovine serum (Gibco Laboratories, USA) supplemented with 100 U/100 ml penicillin/streptomycin (Solarbio, Beijing, China). Cells within the logarithmic growth phase were seeded in 96-well plates at a concentration of 1×10^4^ cells and incubated for 24 h to allow for confluency.

### Preparation of MXD-Medicated Serum

As MXD contains a variety of complex components, including but not limited to tannin and inorganic salts, the experimental results will be interfered if we add MXD into the culture system directly. Besides, although filtered to remove dregs, MXD still contained small particulate matters, which may activate macrophage and interfere with the efficacy of MXD (Wright et al., 1985; Claudio et al., 1995; Voronov et al., 1998; Islam et al., 2011; Higashisaka et al., 2014). Hence, serum pharmacological method was introduced to investigate the effect of MXD on the PM2.5 stimulated inflammatory model in RAW 264.7.

Twenty rats were randomly divided into control group and MXD-medicated group. Rats in the control group were given 1 ml/100 g of saline; rats in the MXD-medicated group were orally administered MXD at the dose of 16.4 g/kg once daily for 5 consecutive days. Blood was collected aseptically via the abdominal aorta 2 h after the last administration and then centrifuged to obtain serum. The serum from the same group were pooled, filtered through 0.22 µm filters, inactivated at 56 °C for 30 min, split, and stored at −70 °C for future use.

### Cell Viability Assay

Cell viability was evaluated with 3-(4,5-Dimethylthiazol-2-yl)-2,5-diphenyltetrazolium bromide (MTT) assay for determining the optimal concentration of PM2.5. Briefly, RAW 264.7 cells (1×10^4^ cells/well) were seeded in 96-well plates. After incubated for 24 h, cells were treated with PM2.5 (1.25, 2.5, 5, 10 and 20 µg/cm^2^) for 24 h. Normally cultured RAW 264.7 cells were used as blank control. Then 20 µl MTT (5 mg/ml) was added to each well for an additional 4 h. The supernatant was removed and the sediment of formazan was dissolved in 200 µl DMSO and shook for 5 min, after which the absorbance value was measured at 570 nm using a microplate reader. The concentration of PM2.5 which was able to decrease the cell viability to about 50% selected for the following experiment.

The optimal concentration (volume fraction expressed as a percentage) of MXD-medicated serum was determined with MTT assay. RAW 264.7 cells (1×10^4^ cells/well) were seeded in 96-well plates and incubated for 24 h. Then cells were incubated with various concentrations of MXD-medicated serum (5% MXD-medicated serum+15% non-immune serum, 10% MXD-medicated serum+10% non-immune serum, 15% MXD-medicated serum+5% non-immune serum, and 20% MXD-medicated serum) for 24 h. Generally, the volume fraction of serum in the culture system doesn’t exceed 20%. Hence, in this experiment, the volume fraction of serum was restricted at 20% for uniformity. Cells in control group were incubated with 20% non-immune serum.

The protective effect of MXD-medicated serum on PM2.5 induced injury in RAW 264.7 cells was assessed using MTT method. After seeded in 96-well plates and incubated for 24 h, cells were divided into control group, PM2.5 group (model group), PM2.5+MXD-medicated serum (5, 10, and 20%) groups. Cells in control group and PM2.5 group were incubated with 20% non-immune serum for 24 h. PM2.5 was added simultaneously after the addition of serum. After 24 h, cell viability was determined as described above.

### Cytokine Assays in Medium

Twenty-four hours after the incubation, the medium was collected and centrifuged at 3,000 g for 10 min to obtain the supernatant. The concentrations of TNF-α, IL-1β, and IL-6 in the culture supernatant were measured by ELISA kits as described above.

### Western Blot Analysis

Isolated lung tissue cubes and RAW 264.7 cells were lysed by radio immunoprecipitation assay (RIPA) lysis buffer containing phenylmethylsulfonyl fluoride (PMSF) (1 mM). The protein concentration was measured by the BCA kit. Equal amount of proteins was separated through 12% SDS polyacrylamide gels and transferred onto polyvinylidene fluoride (PVDF) membranes. The membranes were blocked with 5% nonfat milk at room temperature for 1 h then washed three times and incubated with the primary antibodies (anti-HMGB1, 1:1,000; anti-TLR4, 1:1,000; anti-MyD88, 1:1,000; anti-p-p65, 1:1,000; anti-β-actin, 1:1,000) at 4 °C overnight. The next day, the membranes were washed 3 times for 5 min with TBST and then incubated with the Horseradish peroxidase conjugated secondary antibodies for 2 h at room temperature. Finally, the membranes were washed 3 times for 5 min again. Protein bands were detected by an enhanced chemiluminescence (ECL) detection kit (Wanleibio Co., Ltd., Shenyang, China). The ImageJ software Version 1.41 was used for the quantitative densitometric analysis of the protein bands.

### Verification Experiment Using rHMGB1

For further confirming the protective effect of MXD was at least partially mediated by inhibiting the HMGB1/TLR4/NFκB pathway, a verification experiment using rHMGB1 was conducted. Briefly, cells were divided into four groups: (1) Control group, cells were incubated with 20% nonimmune serum; (2) PM2.5 group, cells stimulated with PM2.5 was incubated with 20% nonimmune serum; (3) Treatment group, cells stimulated with PM2.5 was treated with 20% MXD-medicated serum; (4) Cells stimulated with PM2.5 was treated with 20% MXD medicated-serum plus rHMGB1 (1 µg/ml). The application concentration of rHMGB1 was determined according to the previously published articles (Lv et al., 2009; Han et al., 2016). PM2.5 was added simultaneously after the addition of serum and rHMGB1. After 24 h, the release of cytokines and the expression of TLR4, MyD88 and p-p65 was determined as described in *Cytokine Assays in Medium* and *Western Blot Analysis* section.

### Statistical Analysis

All experimental data were analyzed using SPSS software 22.0 (SPSS Inc., Chicago, IL, USA) and GraphPad Prism 5.0 (GraphPad Software, La Jolla, CA, USA) Statistically significant differences between groups were determined by one-way ANOVA followed by Tukey's post hoc test for multigroup comparisons. A value of *p* < 0.05 was considered statistically significant.

## Results

### Characterization Of MXD Using Mass Spectroscopy

**Figure 1:**
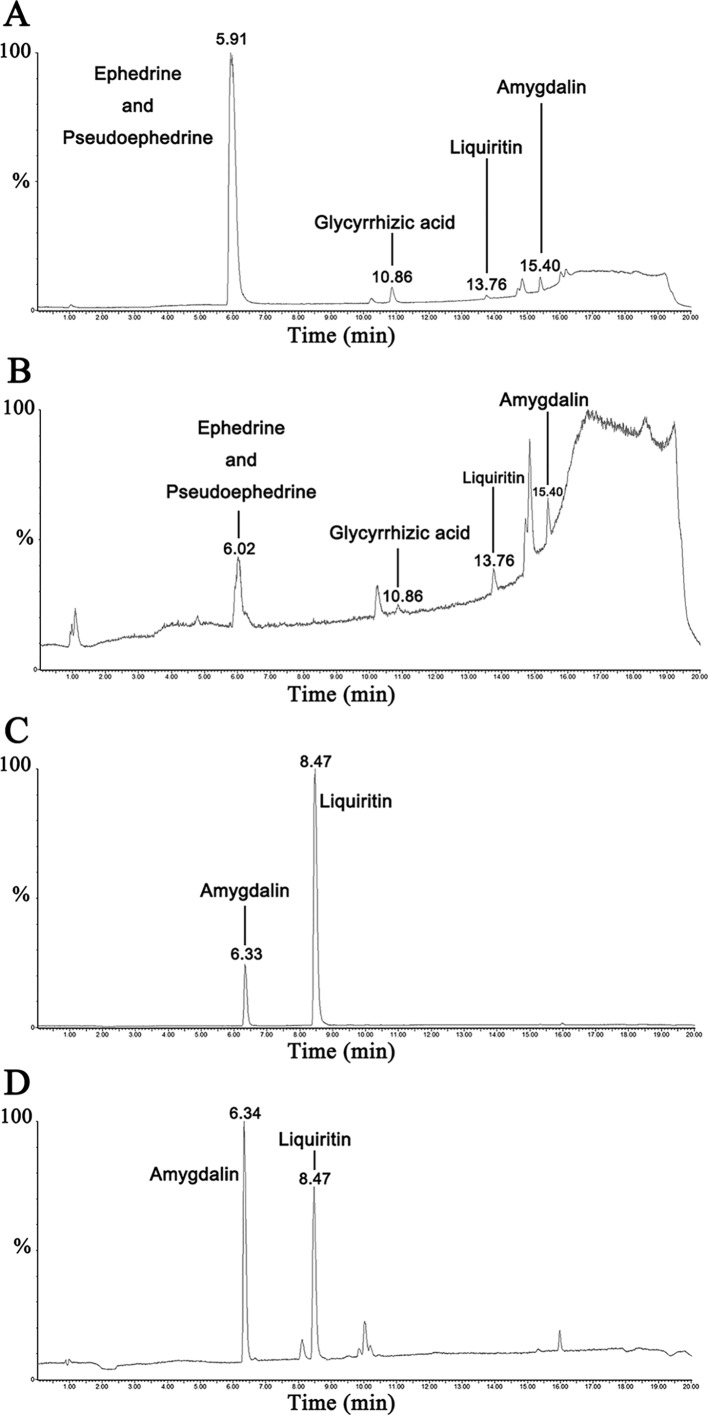
Representative chromatograms of reference standard solution and MXD sample. **(A)** Representative chromatograms of standard solution in positive electrospray ionization mode. **(B)** Representative chromatograms of MXD sample in positive electrospray ionization mode. **(C)** Representative chromatograms of standard solution in negative electrospray ionization mode. **(D)** Representative chromatograms of MXD sample in negative electrospray ionization mode.

**Figure 2:**
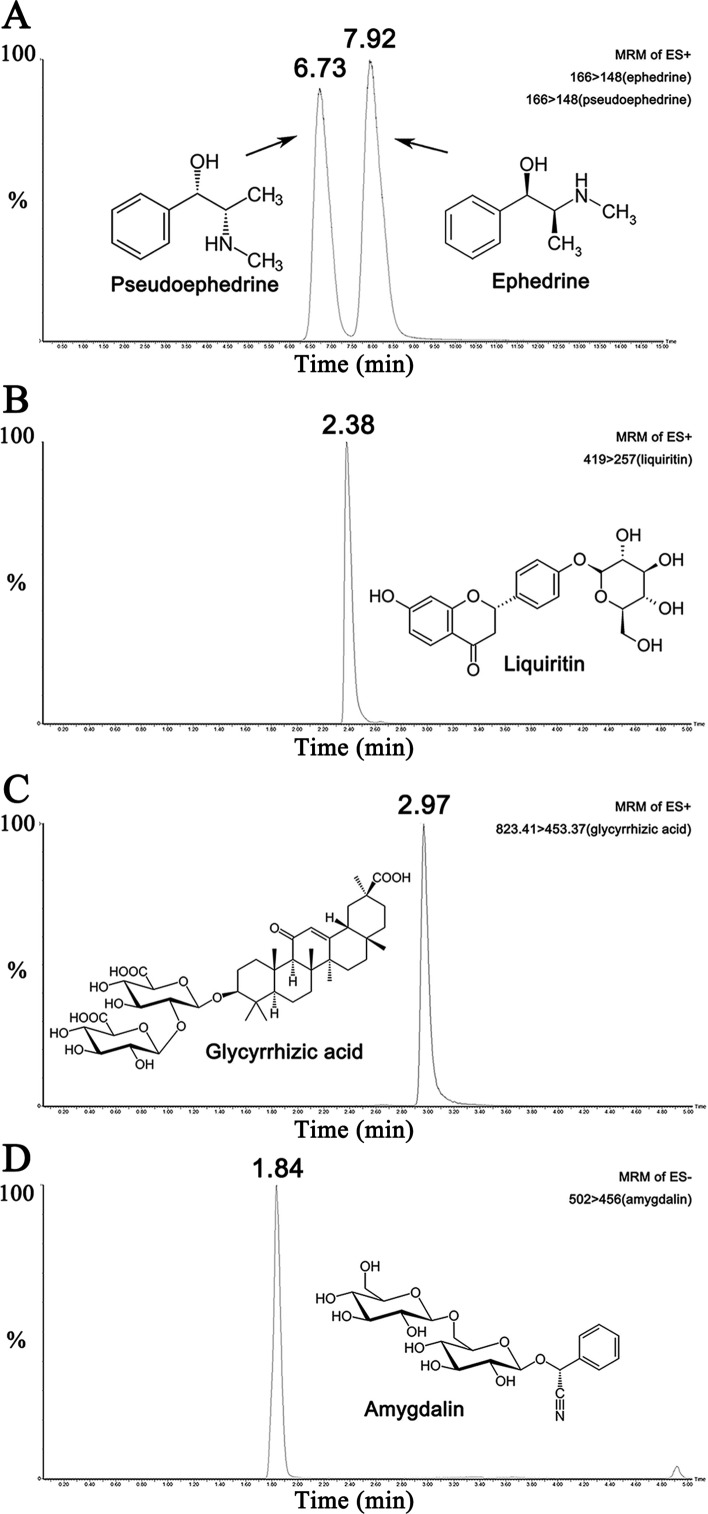
Representative chromatograms of the five compounds in reference standard solution. **(A)** Representative chromatograms of ephedrine (500 ng/ml) and pseudoephedrine (500 ng/ml) in standard solution. **(B)** Representative chromatogram of liquiritin (500 ng/ml) in standard solution. **(C)** Representative chromatogram of glycyrrhizic acid (500 ng/ml) in standard solution. **(D)** Representative chromatogram of amygdalin (500 ng/ml) in standard solution.

**Figure 3:**
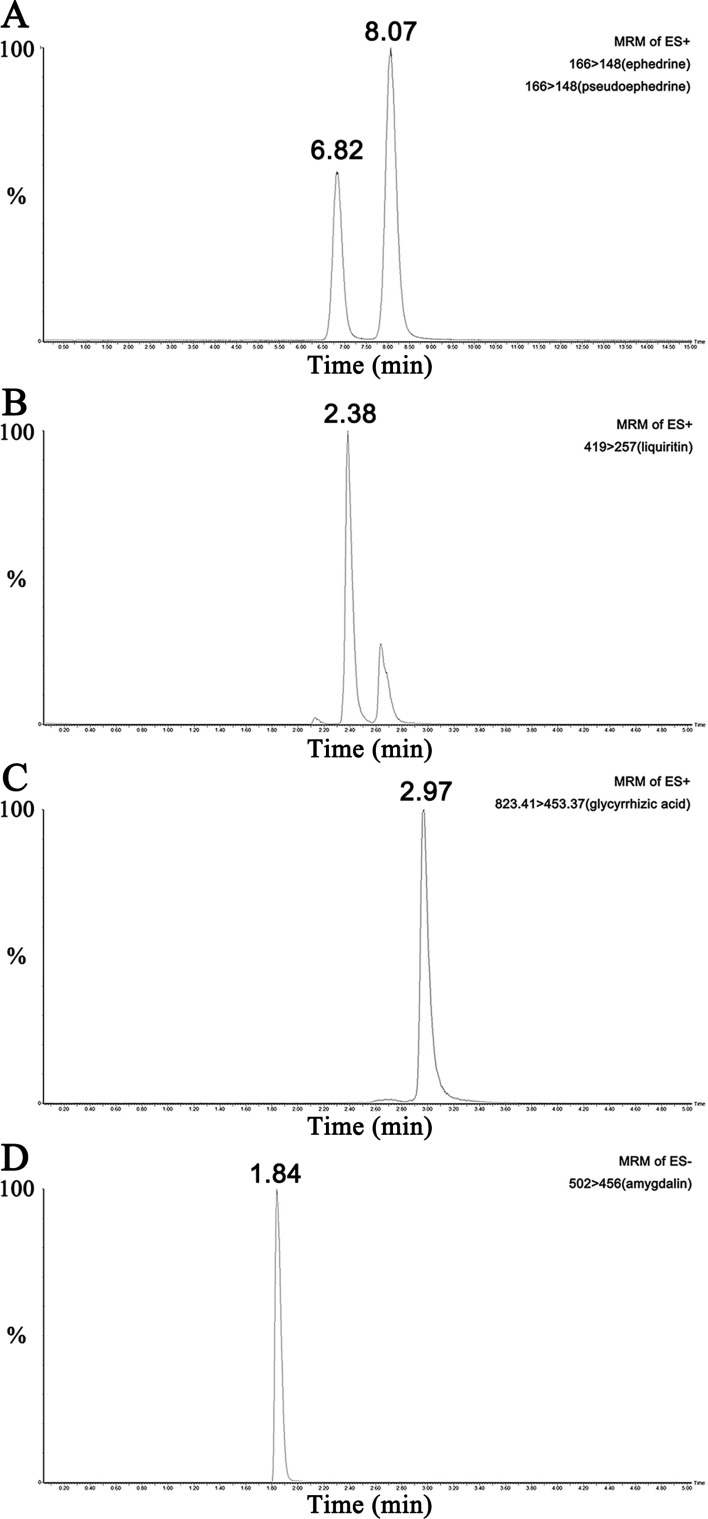
Representative chromatograms of the five compounds in MXD sample. **(A)** Representative chromatograms of ephedrine and pseudoephedrine in MXD sample. **(B)** Representative chromatogram of liquiritin in MXD sample. **(C)** Representative chromatogram of glycyrrhizic acid in MXD sample. **(D)** Representative chromatogram of amygdalin in MXD sample.

UPLC-MS/MS results of reference standard compounds and MXD sample were shown in **Figures 1**–**3**. Five major components of MXD were verified by comparing individual peak retention time and quantifier ion with that of reference standard compounds. External standard method of peak area was employed for quantitative analysis. The concentrations of major components of MXD are 1.135 mg/ml, 0.643 mg/ml, 1.041 mg/ml, 2.019 mg/ml, and 12.280 mg/ml for ephedrine, pseudoephedrine, liquiritin, glycyrrhizic acid, and amygdalin respectively.

### Histopathological Results

H&E staining was used to monitor tissue structures and quantify morphological changes.

**Figure 4:**
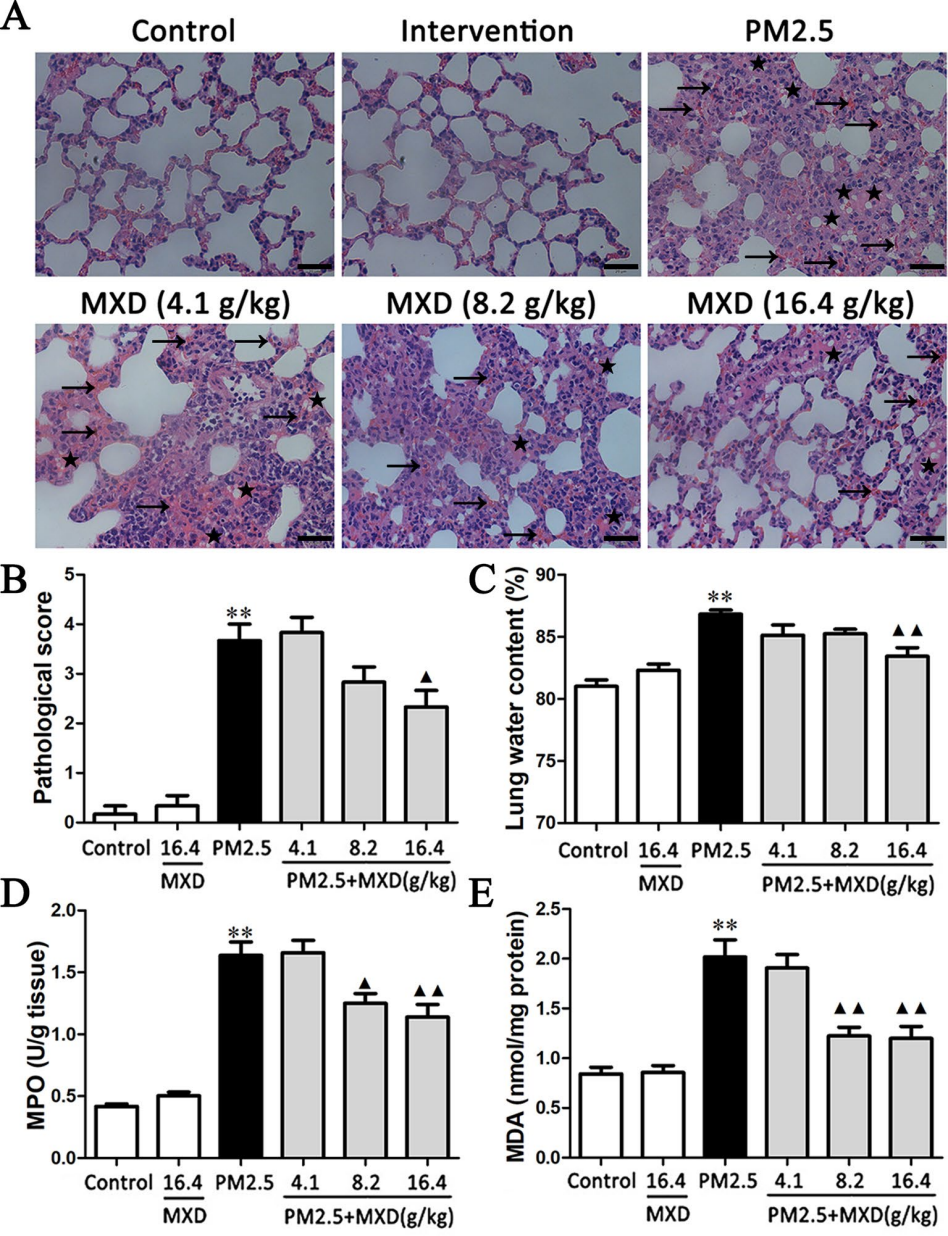
Effects of MXD on PM2.5 induced pathological changes and biochemical indexes changes in lung tissue after a 5-day continuous treatment. **(A)** Effects of MXD on the pathological changes of the lung tissue induced by PM2.5. The local hemorrhage was marked with arrow, and the local edema was marked with lightning bolt. Scale bar, 20 μm (×400). **(B)** Quantified scoring of pathological changes in lung tissue (n = 6). **(C)** Effects of MXD on the lung edema induced by PM2.5 (n = 8). **(D)** Effects of MXD on MPO activity in lung tissues (n=6). **(E)** Effects of MXD on MDA content in lung tissues (n = 7). Data are expressed as mean ± SEM. **p < 0.01 vs. Control group; ^▴^ *p* < 0.05, ^▴▴^ *p* < 0.01 vs. PM2.5 group.

As shown in **Figure 4A**. In control groups, sections of all structures of the lung including bronchioles, alveolus, and pulmonary vessel showed normal morphology. The most severe lung injury was observed in the PM2.5 group. The remarkable changes in the PM2.5 group were the thickening of alveolar walls, lung edema, and alveolar hemorrhage. However, these histopathologic changes were markedly alleviated with the treatment of MXD. The lung damage score was significantly higher in PM2.5 group compared to that of control group (**Figure 4B**, *p* < 0.01). MXD (16.4 g/kg) treatment significantly decreased the lung damage score (*p* < 0.05), indicating that MXD repressed PM2.5 induced histopathological damage of the lung.

### MXD Inhibited PM2.5 Induced Lung Edema

During lung injury, the increased capillary permeability may lead to lung edema. Two hours after the last drug administration, the lung water content was evaluated.

As shown in **Figure 4C**, PM2.5 significantly increased the lung water content in comparison with that of the control group (*p* < 0.01). Treatment with MXD (16.4 g/kg) significantly decreased the lung water content compared with PM2.5 group (*p* < 0.01), indicating that the PM2.5 induced lung edema was attenuated by MXD.

### MXD Reduces PM2.5 Induced MPO Activity and MDA Content

MPO is an index of neutrophil infiltration and MDA content is an indicator of lipid peroxidation, both of which are regarded as an index to evaluate severity of tissue injury.

As shown in **Figure 4D**, PM2.5 stimulation increased the MPO activity in lung tissue compared with the control group (*p* < 0.01). In contrast, the levels of lung MPO activity was down-regulated by MXD (8.2 g/kg and 16.4 g/kg) significantly (*p* < 0.05 and *p* < 0.01).

As shown in **Figure 4E**, PM2.5 stimulation increased the content of MDA significantly (*p* < 0.01), which was reduced significantly by MXD (8.2 g/kg and 16.4 g/kg) (*p* < 0.01).

Our results suggested that MXD can alleviated the severity of tissue injury induced by PM2.5.

### MXD Reduces the Infiltration of CD68 Positive Macrophages in Lung Tissue

The excessive activation of macrophage plays a critical role in the progress of lung injury. CD68 expressed on the surface of macrophage is a well-characterized marker of macrophage activation (Leenen et al., 1994; Dambach et al., 2002; Lloyd et al., 2008; Iwasaki et al., 2011). Hence, the expression of CD68 was detected using IHC to evaluate the activation of macrophage in lung tissue.

**Figure 5:**
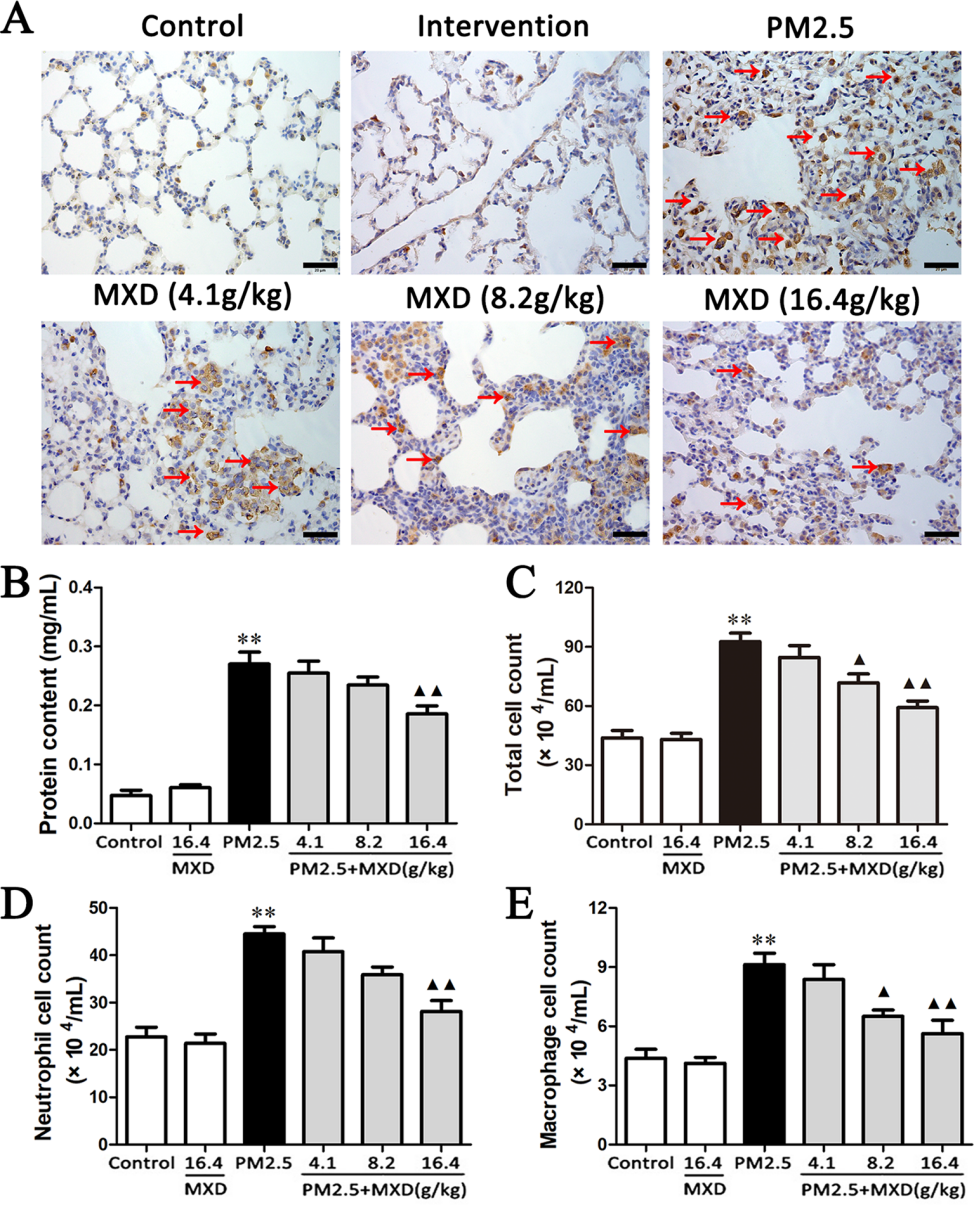
Effects of MXD on PM2.5 induced macrophage activation and alveolar barrier disruption in lung tissue after a 5-day continuous treatment. **(A)** Effects of MXD on the expression of CD68 in lung tissue. CD68 positive cell were marked with arrow. Scale bar, 20 μm (×400). **(B)** Effects of MXD on the protein content in BALF (n = 7). **(C)** Effects of MXD on the total cell count in BALF (n = 8). (D) Effects of MXD on the neutrophil cell count in BALF (n = 8). (E) Effects of MXD on the macrophage cell count in BALF (n = 8). Data are expressed as mean ± SEM. **p < 0.01 vs. Control group; ^▴^ *p* < 0.05, ^▴▴^ *p* < 0.01 vs. PM2.5 group.

As shown in **Figure 5A**, the accumulation of CD68 positive macrophages in lung tissue was significantly increased in PM2.5 group compared with that of control group. After treatment with MXD, the number of CD68 positive macrophage was significantly reduced, indicating that MXD attenuated the PM2.5 induced activation of macrophage in lung tissue.

### MXD Alleviates PM2.5 Induced Alveolar Permeability

Severe injury to the alveolar epithelial/endothelial membrane may result in the disruption of alveolar capillary barrier, which is manifested by the leakage of fluid from the vascular circulation into the interstitial and alveolar spaces. The leakage of fluid will result in increased protein concentration and cell number in BALF. Hence, the total protein content and cell number in BALF were detected to evaluate the effect of MXD on lung epithelial barrier function.

As shown in **Figure 5B**, protein content was significantly increased in PM2.5 group compared with that of control group (*p* < 0.01). Treatment with MXD (16.4 g/kg) showed a significant decrease in protein content in comparison with that of PM2.5 group (*p* < 0.01).

As shown in **Figure 5C**, after PM2.5 stimulation, the total cell count in BALF was significantly increased (*p* < 0.01), which was significantly reversed by the treatment of MXD (8.2 g/kg and 16.4 g/kg) significantly (*p* < 0.05 and *p* < 0.01). For further confirming what type of inflammatory cell is elicited by PM2.5 challenge, the number of neutrophils and macrophages in the BALF was counted respectively using Wright-Giemsa staining. As can be seen in **Figures 5D, E**, stimulation with PM2.5 increased the number of both neutrophils and macrophages in BALF significantly (*p* < 0.01). Treatment with MXD (16.4 g/kg) decreased the cell count of both neutrophils and macrophages (*p* < 0.01).

Taken above, our results showed MXD can alleviate the PM2.5 induced alveolar capillary barrier disruption. Notably, the infiltration of inflammatory cells, such as neutrophils and macrophages, was also reduced.

### MXD Attenuates PM2.5 Induced Inflammatory Cytokine Production in Serum and BALF

Inflammatory factors contribute to the development of lung injury, among which TNF-α, IL-1β and IL-6 are the major cytokines (Quay et al., 1998; Smith et al., 1998; Koksoy et al., 2001; Goodman et al., 2003; Saito et al., 2008; Wilson et al., 2010; Pedroza et al., 2011; Patel et al., 2013). Hence, the concentrations of TNF-α, IL-1β and IL-6 in both serum and BALF were determined using ELISA kits.

**Figure 6:**
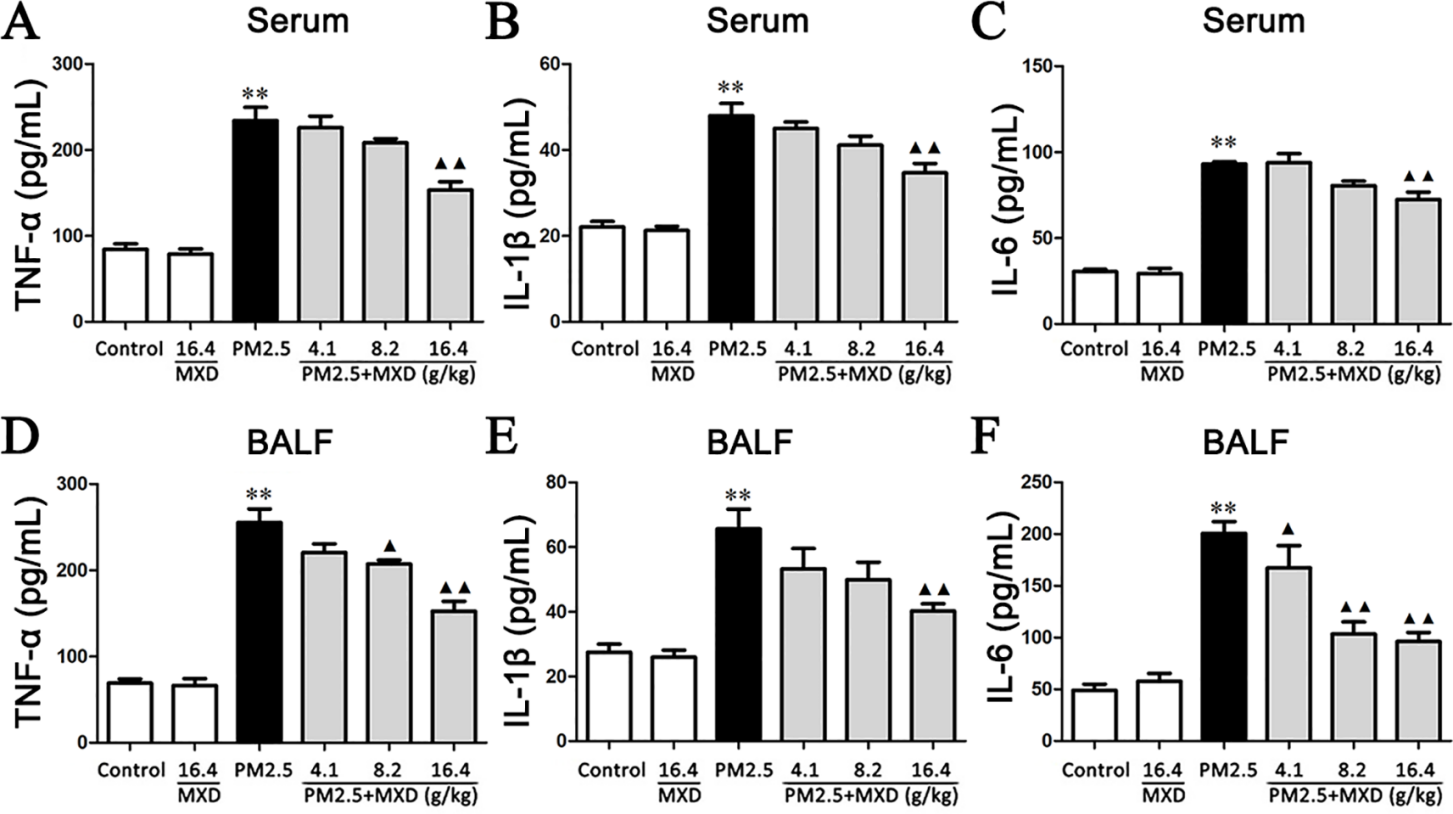
Effects of MXD on the levels of proinflammatory factors in serum and BALF after a 5-day continuous treatment. The levels of TNF-α **(A)**, IL-1β **(B)** and IL-6 **(C)** in serum were examined. The levels of TNF-α **(D)**, IL-1β **(E)**, and IL-6 **(F)** in BALF were examined. Data are expressed as mean ± SEM, n = 5. **p < 0.01 vs. Control group; ^▴^ *p* < 0.05, ^▴▴^ *p* < 0.01 vs. PM2.5 group.

As shown in **Figures 6A–C**. The effect of MXD on TNF-α, IL-1β and IL-6 production in serum was analyzed by ELISA after PM2.5 challenge. Compared with the control group, the levels of TNF-α, IL-6, and IL-1β in serum were significantly increased after PM2.5 stimulation (*p* < 0.01). MXD (16.4 g/kg) reduced TNF-α (*p* < 0.01), IL-1β (*p* < 0.01), and IL-6 (*p* < 0.01) production compared with that of the PM2.5 group.

As shown in **Figures 6D–F**, PM2.5 exposure resulted in a marked increase in the levels of TNF-α, IL-1β and IL-6 in BALF compared with control group (*p* < 0.01). Treatment with MXD (16.4 g/kg) significantly ameliorated the levels of these inflammatory cytokines (*p* < 0.01).

Our results showed that treatment with MXD reduced the release of pro-inflammatory cytokines after PM2.5 challenge, indicating the anti-inflammatory potential of MXD.

### Effect of MXD on Cell Viability of RAW 264.7 Exposed to PM2.5

**Figure 7:**
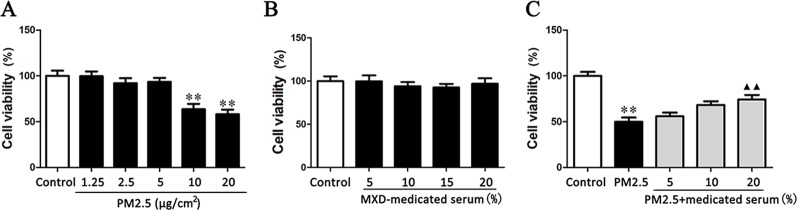
Effects of PM2.5 and MXD-medicated serum on the cell viability of RAW 264.7 after a 24-hour incubation. **(A)** The cytotoxic effects of PM2.5 on RAW 264.7 cell viability. **(B)** The confirmation for the non-cytotoxic concentrations (volume fractions) of MXD-medicated serum. **(C)** Protective effect of MXD-medicated serum on RAW 264.7 exposed to PM2.5. Data are expressed as mean ± SEM, n = 6. **p < 0.01 vs. Control group; ^▴▴^ *p* < 0.01 vs. PM2.5 group.

In order to assess the effect of MXD mediated serum on PM2.5 induced injury in RAW 264.7 cells, we first confirmed the occurrence of PM2.5 induced damage by comparing the viability between the PM2.5-stimulated group and the control group. As shown in **Figure 7A**, when exposed to PM2.5 at 10 µg/cm^2^ for 24 h, the cell viability of RAW 264.7 was significantly decreased by 50% in comparison with normal cultured cells (*p* < 0.01). Hence, the concentration of PM2.5 at 10 µg/cm^2^ was selected for the following experiment.

Then the non-cytotoxic concentrations (volume fractions) range of MXD-medicated serum was determined by incubating RAW 264.7 cells with MXD-medicated serum at a series of concentrations. As shown in **Figure 7B**, MXD-medicated serum at the concentrations from 5 to 20% showed no effect on the cell viability (*p* > 0.05). Considering the volume fraction of serum in culture system doesn’t exceed 20% generally, the concentrations of MXD-medicated serum at 5, 10 and 20% were applicable to all subsequent experiments.

The protective effect of MXD-medicated serum on RAW264.7 cell challenged by PM2.5 was evaluated based on the selected PM2.5 concentration and MXD-medicated serum concentrations. As shown in **Figure 7C**, MXD-medicated serum at the concentration of 20% significantly reversed the decrease of cell viability induced by PM2.5 (*p* < 0.01).

### Effect of MXD on the Production of Proinflammatory Cytokines *In Vitro*

**Figure 8:**
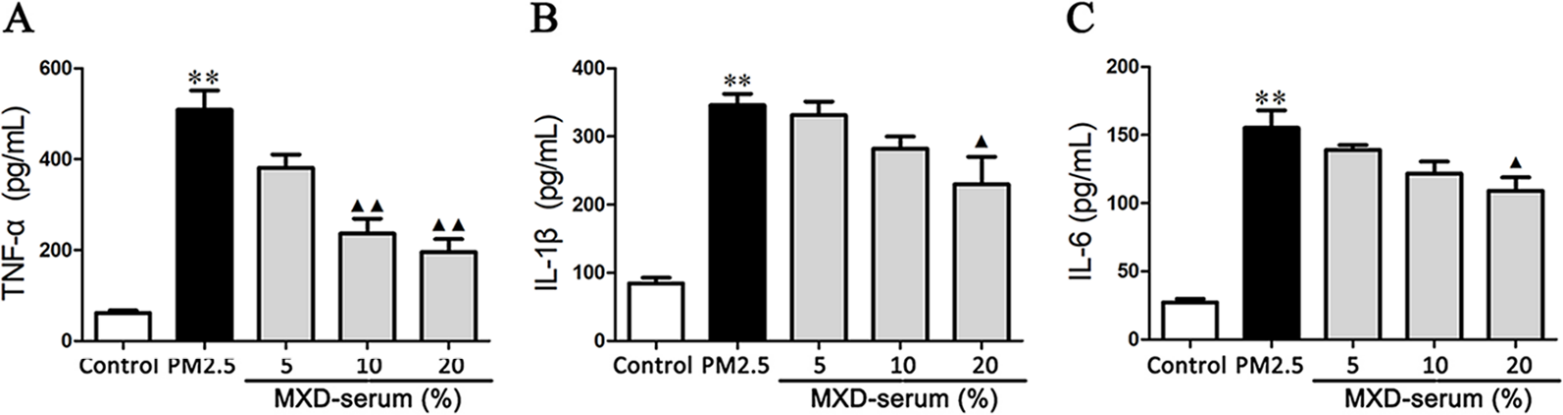
Effects of MXD-medicated serum on the secretion of proinflammatory factors from RAW 264.7 after a 24-hour incubation. The levels of TNF-α **(A)**, IL-1β **(B)**, and IL-6 **(C)** in the culture supernatant were examined. Data are expressed as mean ± SEM, n = 5. **p < 0.01 vs. Control group; ^▴^ *p* < 0.05, ^▴▴^ *p* < 0.01 vs. PM2.5 group.

The effects of MXD-medicated serum on TNF-α, IL-1β, and IL-6 production in the culture supernatant were detected by ELISA. As shown in **Figures 8A–C**, PM2.5 challenge increased the level of TNF-α, IL-1β, and IL-6 compared with the control group (*p* < 0.01). In contrast, incubation with 20% MXD-medicated serum decreased the levels of TNF-α (*p* < 0.01), IL-1β (*p* < 0.05), and IL-6 (*p* < 0.05), indicating the inhibitory effect of MXD-medicated serum on the release of proinflammatory cytokines.

### Effect of MXD on PM2.5 Induced HMGB1/TLR4/NFκB Activation *In Vivo* and *In Vitro*

Recent studies suggest that HMGB1 interacts with TLR4 and induces the activation of NFκB signaling pathway, which plays a major role in the release of inflammatory cytokines and the development of organ dysfunction (Yang et al., 2014; Sun et al., 2015). Hence, the expressions of the key proteins in HMGB1/TLR4/NFκB signaling pathway were examined by Western blot both *in vivo* and *in vitro*.

**Figure 9:**
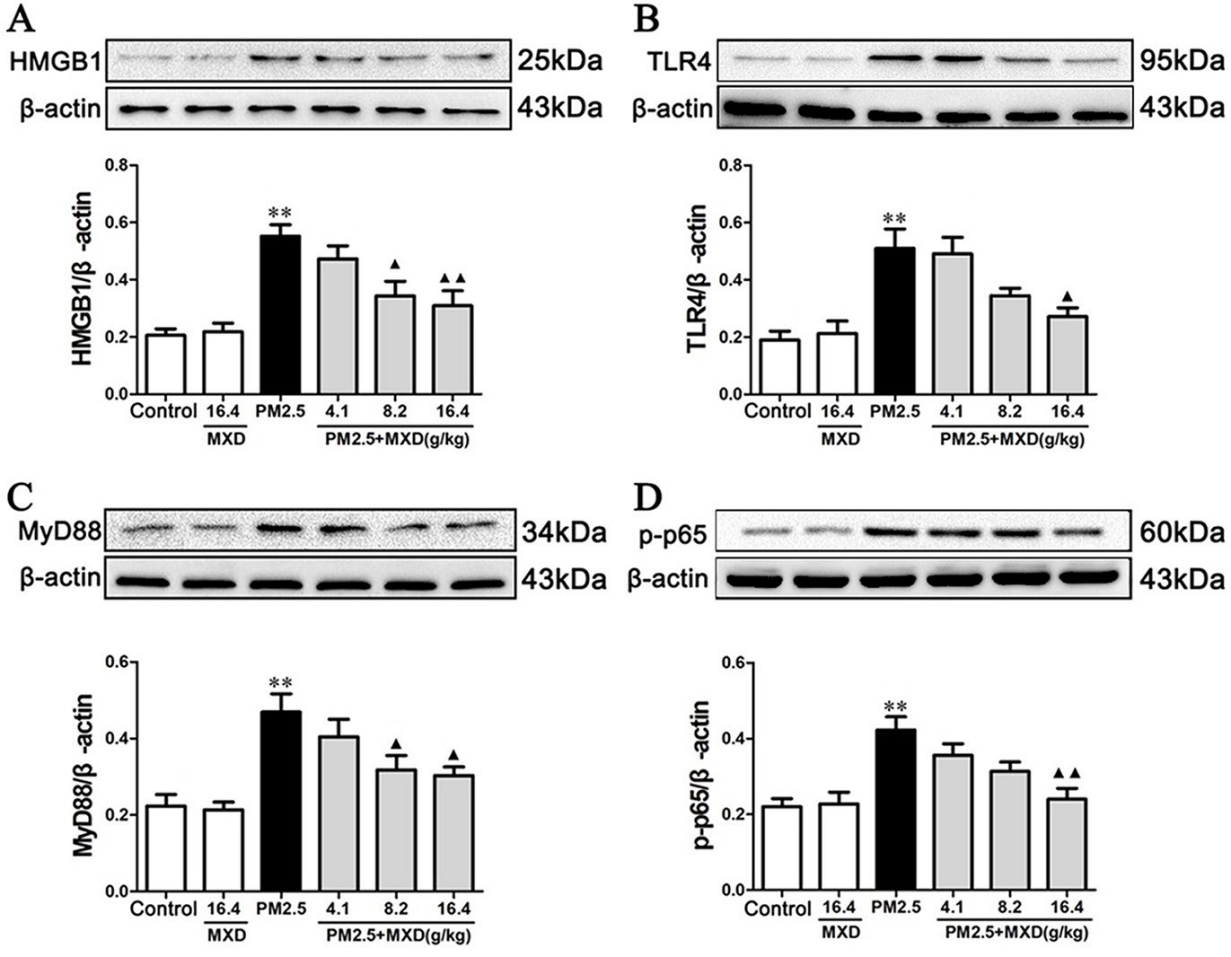
Effects of MXD on the protein levels of HMGB1 **(A)**, TLR4 **(B)**, MyD88 **(C)**, and p-p65 **(D)** in rat lung tissues after a 5-day continuous treatment. The values presented are the mean ± SEM (n = 5) of three independent experiments. The density values of protein expression were normalized to β-actin. **p < 0.01 vs. Control group; ^▴^ *p* < 0.05, ^▴▴^ *p* < 0.01 vs. PM2.5 group.

As shown in **Figure 9**, the protein levels of HMGB1, TLR4, MyD88, and p-p65 in lung tissues were significantly increased in PM2.5 group compared with control group (*p* < 0.01), while treatment with MXD (16.4 g/kg) significantly decreased the protein levels of HMGB1 (*p* < 0.01), TLR4 (*p* < 0.05), MyD88 (*p* < 0.05), and p-p65 (*p* < 0.01).

**Figure 10:**
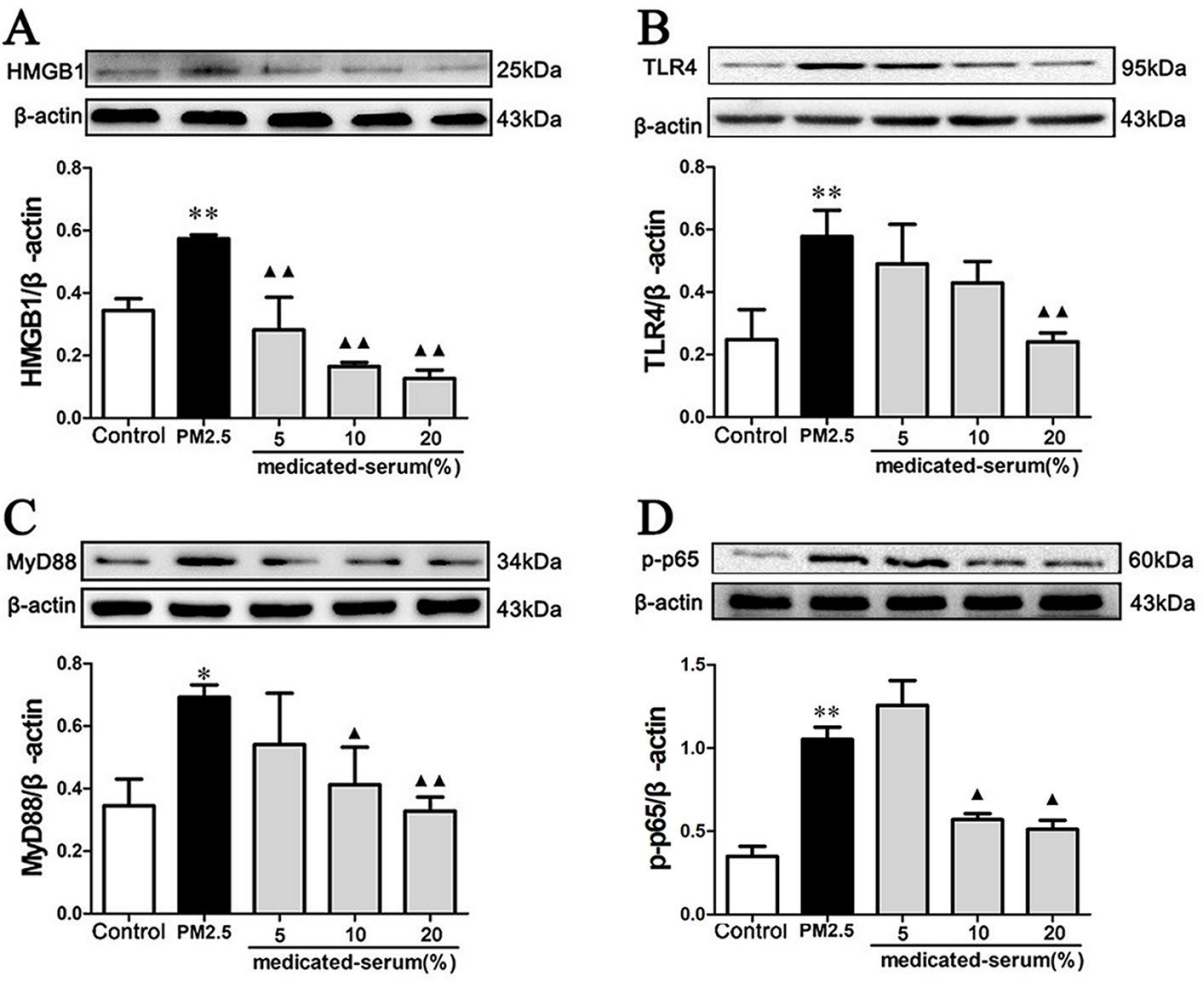
Effects of MXD-medicated serum on the protein levels of HMGB1 **(A)**, TLR4 **(B)**, MyD88 **(C)**, and p-p65 **(D)** in RAW 264.7 cells after a 24-hour incubation. The values presented are the mean ± SD (n = 3) of three independent experiments. The density values of protein expression were normalized to β-actin. *p < 0.05, **p < 0.01 vs. Control group; ^▴^ *p* < 0.05, ^▴▴^ *p* < 0.01 vs. PM2.5 group.

As shown in Figure 10, stimulation with PM2.5 significantly up-regulated the proteins levels of HMGB1, TLR4, MyD88, and p-p65 in RAW 264.7 cells (*p* < 0.01). Incubation with 20% MXD-medicated serum significantly down-regulated the expressions of HMGB1 (*p* < 0.01), TLR4 (*p* < 0.01), MyD88 (*p* < 0.01), and p-p65 (*p* < 0.05), which was in accordance with what we observed *in vivo*.

### The Effect of MXD-Medicated Serum Was Reversed by rHMGB1

**Figure 11:**
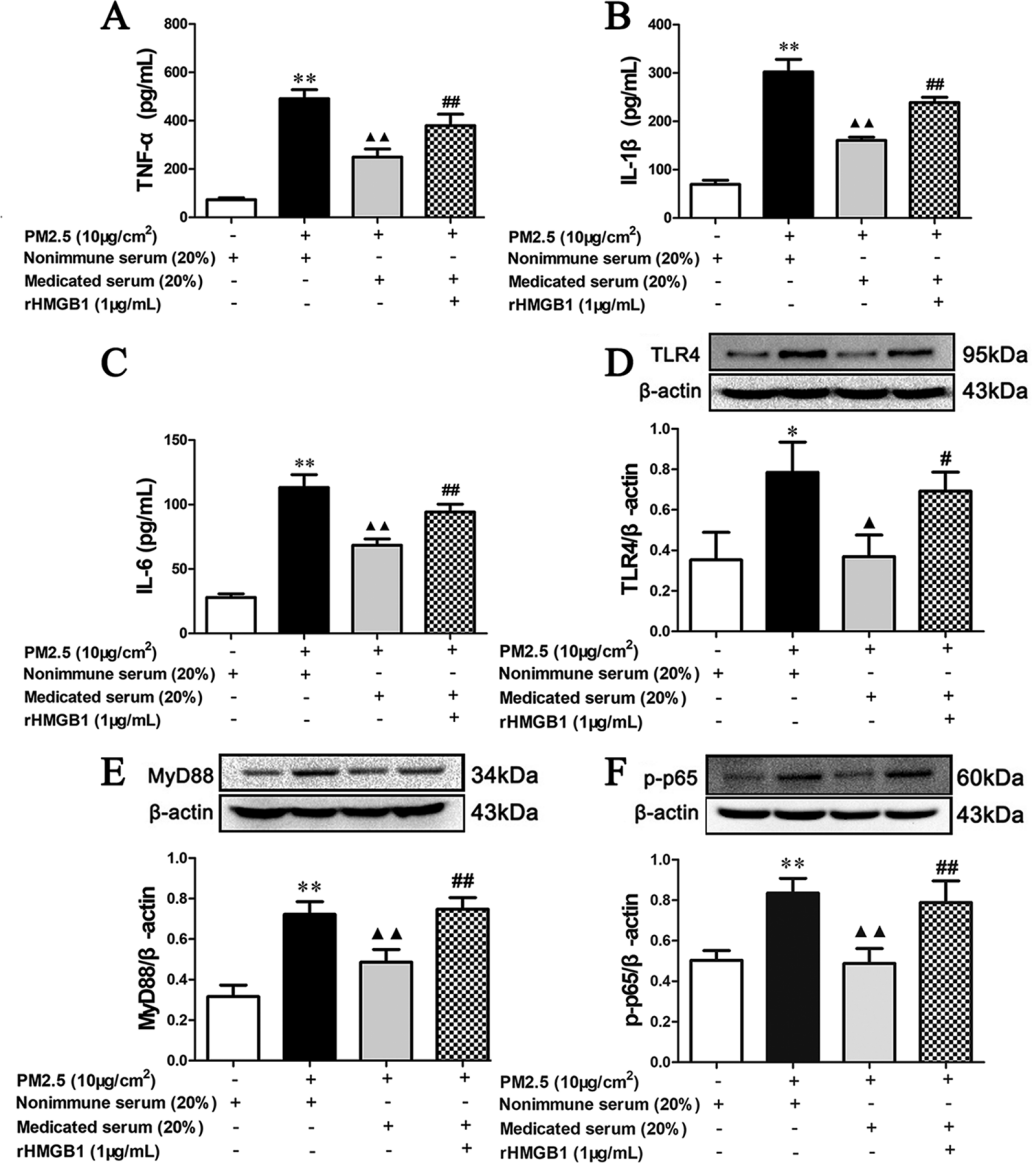
Effects of MXD-medicated serum with or without rHMGB1 treatment on the PM2.5 induced release of inflammatory cytokines and the activation of HMGB1/TLR4/NFκB pathway in RAW 264.7 after a 24-hour incubation. The concentration of TNF-α **(A)**, IL-1β **(B)**, and IL-6 **(C)** in the supernatant. The expression of TLR4 **(D)**, MyD88 **(E)**, and p-p65 **(F)** in RAW264.7 cell. For ELISA assay, data are expressed as mean ± SEM, n = 5. For Western blot, the values presented are the mean ± SD (n = 3) of three independent experiments. The density values of protein expression were normalized to β-actin. *p < 0.05, **p < 0.01 vs. Control group; ^▴^ *p* < 0.05, ^▴▴^ *p* < 0.01 vs. PM2.5 group; ^#^ *p* < 0.05, ^##^ *p* < 0.01 vs. Treatment group.

As shown in **Figures 11A–C**, 20% MXD-medicated serum significantly inhibited the release of proinflammatory factors (*p* < 0.01), which was consistent with our results above. Notably, co-incubation with rHMGB1 abolished the inhibitory effects of MXD-medicated serum on the release of TNF-α (*p* < 0.01), IL-1β (*p* < 0.01), and IL-6 (*p* < 0.01).

As shown in **Figures 11D–F**, in accordance with our results above, 20% MXD-medicated serum reduced the expression of TLR4 (*p* < 0.01), MyD88 (*p* < 0.01), and p-p65 (*p* < 0.01). The regulatory effect was again reversed in MXD-medicated serum plus rHMGB1 group (*p* < 0.01).

These results demonstrate that the incubation of MXD-medicated serum significantly alleviates inflammatory reaction caused by PM2.5 in RAW264.7 cell by antagonizing the function of HMGB1 and the mechanism involved in the regulation of downstream TLR4/MyD88/NFκB signaling pathway.

## Discussion

**Figure 12:**
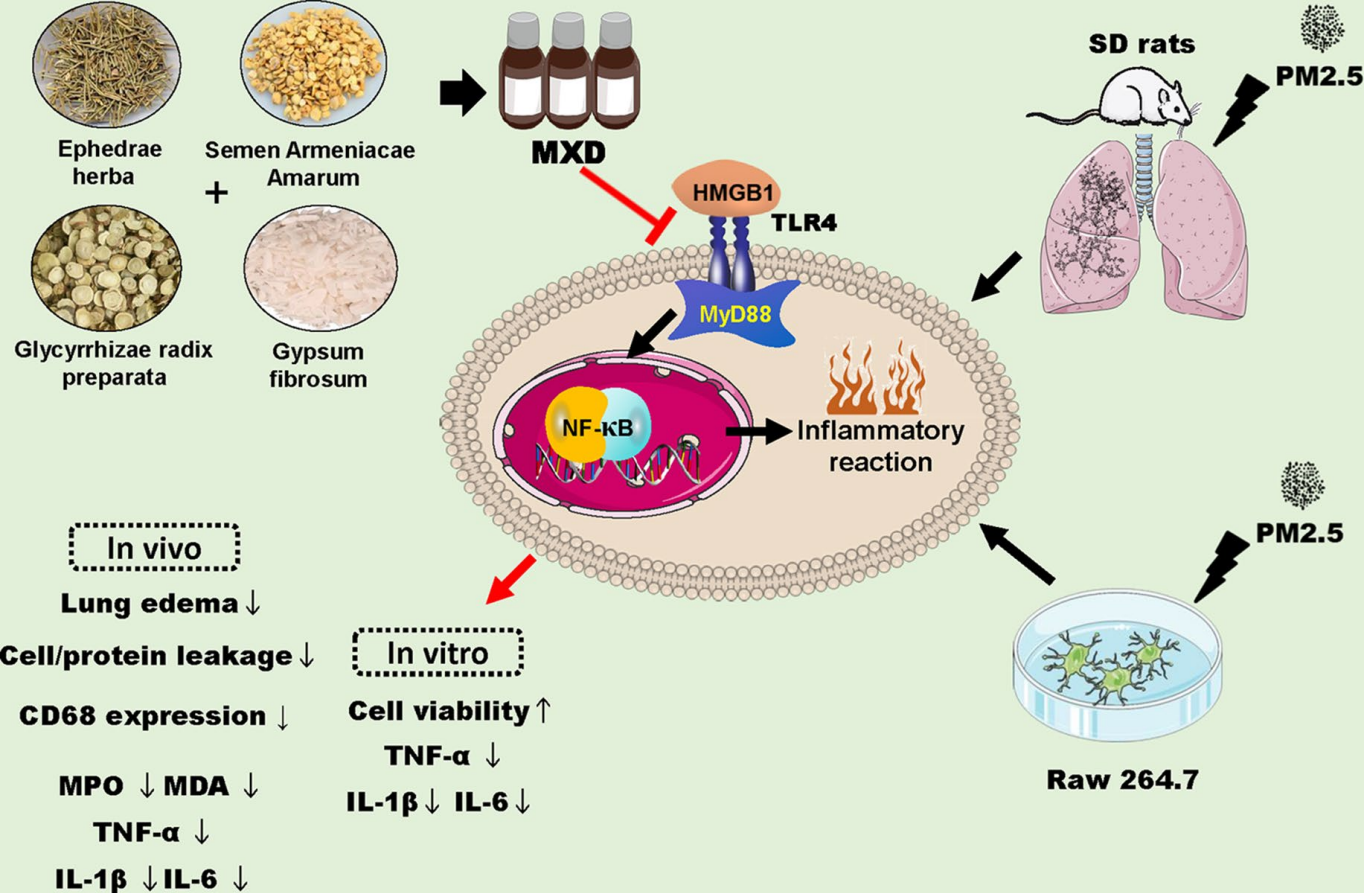
Schematic diagram depicting proposed mechanisms for the protection of MXD against PM2.5 induced injury. MXD decreased the expression of HMGB1, TLR4 and downstream MyD88, and inhibited NFκB activation. In addition, the downregulation of proinflammatory cytokines was involved in the protection of MXD. Red arrow and perpendicular line: the effects of MXD.

The present study demonstrated that treatment with MXD (or MXD-medicated serum) could protect against PM2.5 induced injury both *in vivo* and *in vitro*. MXD could inhibit activation of the HMGB1/TLR4/NFκB signaling pathway and decreased inflammatory cytokine level thereby attenuating inflammatory injury (**Figure 12**).

With the rapid development of modern society, the environment has been seriously polluted. Currently, most northern China areas are deeply trapped in the haze, and the main contaminant of the air is PM (Huang et al., 2014; Yang et al., 2017). PM is composed of inorganic matters, organic matters, metallic minerals, bacteria, and a variety of complex ingredients (Querol et al., 2004; Kleindienst et al., 2010; Huang et al., 2014; Pipal et al., 2014). A most harmful particle to human health is PM2.5. Several studies have shown that PM2.5 can travel deeper into the lungs, and exposure to PM2.5 is associated with pulmonary and cardiovascular diseases and cancer (Vineis and Husgafvel-Pursiainen, 2005; Tecer et al., 2008; Polichetti et al., 2009; Silva et al., 2010; Leiva G et al., 2013; Martínez-Cinco et al., 2016; Xing et al., 2016). During the process of pulmonary diseases, inflammatory reaction plays an important role (Welbourn and Young, 1992; Ghio and Devlin, 2001; Ricard et al., 2001; Peng et al., 2004; Menezes et al., 2005). Many preliminary studies confirmed the anti-inflammatory and immune-regulatory effects of natural products, while several data showed a decreased effect of their metabolites on cytokines for inflammation, such as IL-1, IL-6, and TNF-α (Teng et al., 2016, Teng et al., 2017; Chen et al., 2018, Chen et al., 2019; Gupta et al., 2019; Magalhães et al., 2019; Saudagar and Saokar, 2019; Su et al., 2019; Zhang et al., 2019). Hence, it arouses our interest that whether MXD, a famous traditional Chinese medicine formula widely used in respiratory diseases, can alleviate PM2.5 induced lung injury.

Ma huang, a traditional Chinese herb, is often used to treat asthma, nose and lung congestion, and fever with anhidrosis. The main constituent of ma huang is ephedrine and pseudoephedrine, which showed potential in attenuating inflammatory response (Kasahara et al., 1985; Zheng et al., 2012; Wu et al., 2014).

Ku xing ren is widely used for treatment of cough and asthma. Modern pharmacology indicates that amygdalin is an effective component of ku xing ren (Gao et al., 2012). Recent studies have indicated that amygdalin showed anti-inflammatory activity both *in vivo* and *in vitro* (Yang et al., 2007; Lin and Lin, 2011).

Shi gao is often used in combination with Ma huang to exert the antipyretic and anti-asthmatic activities (Mei et al., 2016).

Gan cao is one of the oldest and most popular herbal medicines in the world, and is recorded in the pharmacopoeias of many Asian and European countries including China, Japan, the United Kingdom and others (Zhang and Ye, 2009). The main constituent of gan cao is glycyrrhizic acid which exhibits anti-inflammatory, anti-viral, anti-allergenic, anti-ulcer, and anti-oxidative properties (Gan et al., 2012; Ming and Yin, 2013; Tang et al., 2015). Recent studies have shown that glycyrrhizic acid on the regulation of HMGB1/TLR4/NFκB signaling pathway in many diseases (Lau et al., 2014; Pang et al., 2016).

At present, the most commonly used approach for establishing an animal model of PM2.5 induced lung injury is intratracheal instillation of PM2.5 suspension (Li et al., 2013; Dysart et al., 2014). In this study, we establish the model of acute lung injury by instillation with PM2.5 suspension.

As one of the major characteristics of lung injury, the magnitude of pulmonary edema was quantified by evaluating the lung water content (Liu et al., 2013; Feng et al., 2016). Our results showed that PM2.5 induced edema of the lung, which was reflected by the significant increase in lung water content. Administration of MXD ameliorated PM2.5 induced lung edema.

MPO activity, a marker reflecting the parenchymal infiltration of neutrophils, was assessed as an important index of tissue damage (Lin et al., 2016; Zhou et al., 2016). Increased MPO level may strengthen ability to resist infection, however, it may also increase the synthesis of hypochlorous acid, which can be detrimental to normal tissue (Wei et al., 2004). Overall, the results suggest that MXD attenuates PM2.5 induced elevated MPO level. MDA, a marker of lipid peroxidation, is usually measured as a tissue lesion index. After PM2.5 stimulation, MDA was significantly increased, which was significantly reversed with the treatment of MXD, indicating the effect of MXD against oxidative damage.

Alveolar macrophages have been reported to play important roles in PM2.5 induced lung injury (Meng and Zhang, 2007; Xing et al., 2016). Stimulating alveolar macrophages with PM2.5 induce the production of inflammatory mediators, which contribute to the severity of lung injury by initiating, amplifying, and perpetuating the inflammatory response (Becker et al., 1996; Ning et al., 2000; Hetland et al., 2005). These cytokines not only amplify the inflammatory cascade and cause the inflammatory injury, but also recruit neutrophils into lung and exhibit increased MPO activity in the lungs (Xie et al., 2009). In the present study, increased levels of TNF-α, IL-1β, and IL-6 in serum, BALF and RAW 264.7 cell culture supernatant have been noted in PM2.5 groups. MXD significantly inhibited the production of TNF-α, IL-1β, and IL-6, which indicated that the protective effects of MXD on PM2.5 induced lung injury may be attributed to the inhibition of inflammatory cytokines.

HMGB1 is a highly conserved DNA binding protein. As a nuclear protein, HMGB1 acts as an architectural chromatin-binding factor that bends DNA and promotes protein assembly on specific DNA targets (Scaffidi et al., 2002). In addition to its intranuclear role, HMGB1 also functions as an extracellular signaling molecule during inflammation (Yang et al., 2010a). HMGB1 can be either actively secreted to the extracellular through the activation of macrophages and monocytes or passively released from necrotic or apoptotic cells (Rovere-Querini et al., 2004; Bell et al., 2006). Extracellular HMGB1 activates multiple membrane receptors, including but not limited to receptor for advanced glycation end products, and TLR4, through which it contributes to the pathogenesis of lung injury, stroke, cancer, and so on (Andersson and Erlandsson-Harris, 2004; Kokkola et al., 2005; Muhammad et al., 2008; Sims et al., 2009; Yang and Tracey, 2010; Yang et al., 2010b; Schierbeck et al., 2011; Tolle and Standiford, 2013; Lv et al., 2016; Pandolfi et al., 2016). Furthermore, extracellular HMGB1 is responsible for the progression of PM2.5 induced lung injury (Shoenfelt et al., 2009; Jing et al., 2017; Tang et al., 2018).

TLR4 locating on the cell plasma membrane is one of the best characterized TLRs, which recognizes the Gram-negative product LPS and participates in the recognition of several endogenous ligands including HMGB1 and heat shock protein (Erridge, 2010; Kay et al., 2014). As a canonical downstream adaptor, MyD88 is closely involved in inflammatory signaling pathways mediated by the TLRs. The downstream NFκB is a critical factor in regulating both innate and adaptive immunity, which enhances various gene expressions during inflammatory responses (Chen et al., 2016). HMGB1 binds with TLR4, leading to nuclear factor-kappa B (NFκB) activation recognized by p65 phosphorylation, and promotes the release of inflammatory factors including TNF-α, IL-1β, and IL-6 (Lawrence, 2009; Tang et al., 2009; Shin et al., 2016). Pharmacological inhibition of TLR4/MyD88/NFκB pathway led to decrease of inflammatory cytokines and showed protective effects on acute lung injury (Jiang et al., 2015). In this study, the expression of HMGB1, TLR4, MyD88, and p-p65 were detected by Western blot analysis *in vivo* and *in vitro*. The results showed that PM2.5 significantly increased the expression of HMGB1, TLR4 and MyD88, and upregulated the phosphorylation level of p65. However, treatment with MXD decreased expression of HMGB1, TLR4 and MyD88 and inhibited the phosphorylation of p65. In all, these results showed that MXD could inhibit the activation of HMGB1/TLR4/NFκB pathway, alleviate the inflammation reaction and protect the lung from PM2.5 induced injury.

However, several limitations of our study must be acknowledged. This study only focuses on the HMGB1/TLR4/NFκB pathway, other pathways related to inflammation such as JAK/STAT and MAPK should be explored in future studies. Moreover, whether the anti-inflammatory effect of MXD requires five components together still remains unknown. Fangjiomics, a recently emerged new discipline to systematically study myriad compatible combinations that may act through multiple targets and modes of actions, maybe a good choice to elucidate the underlying mechanism (Wang et al., 2013; Duan et al., 2015).

In summary, the present study demonstrated that MXD protected against PM2.5 induced lung injury by inhibiting pulmonary edema, MPO activity, MDA content, inflammatory cytokines (IL-1β, IL-6, and TNF-α) production. The protective mechanism of MXD may be related to the inhibition of HMGB1/TLR4/NFκB signaling pathway.

## Data Availability Statement

All datasets generated for this study are included in the article/**Supplementary Material**.

## Ethics Statement

The animal study was reviewed and approved by Institutional Animal Care and Use Committee of China Pharmaceutical University.

## Author Contributions

Participated in research design: Y-xF and Y-fW. Methodology: Y-xF, G-HR, and B-WW. Formal analysis: Y-xF, BZ, Q-yY, and Y-yQ. Conducted experiments: Y-xF, BZ, Q-yY, and Y-yQ. Writing original draft preparation: Y-xF. Review and editing: Y-xF, Y-fW, and Y-mL. Supervision: Y-fW, W-rF, and Y-mL. Project administration: Y-mL. Funding acquisition: Y-fW.

## Funding

This research was funded by the “Key Project of Nanjing Department of Health,” grant number ZKX17047.

## Conflict of Interest

The authors declare that the research was conducted in the absence of any commercial or financial relationships that could be construed as a potential conflict of interest.

## Abbreviations

MXD, Ma Xing Shi Gan Decoction; PM2.5, Particulate matters with diameter of less than 2.5 µm; MPO, Myeloperoxidase; MDA, Malondialdehyde; HE Hematoxylin and eosin; BALF, Bronchoalveolar lavage fluid; IHC, Immunohistochemistry; TNF-α, Tumor necrosis factor-alpha; IL-1β, Interleukin-1beta; IL-6, Interleukin-6; ELISA, Enzyme linked immunosorbent assay; HMGB1, High-mobility group box 1; rHMGB1, Recombinant high-mobility group box 1; TLR4, Toll-like receptor 4; MyD88, Myeloid differentiation factor 88; p-p65, Phosphorylated p65; UPLC-MS, Ultra-performance liquid chromatography-tandem mass spectrometry; PBS, Phosphatic buffer solution; TMB, 3,3′,5,5′-tetramethylbenzidine; RIPA, Radio immunoprecipitation assay; PMSF, Phenylmethylsulfonyl fluoride; BCA, Bicinchoninic acid; PVDF, Polyvinylidene fluoride; ECL, Enhanced chemiluminescence; NFκB, Nuclear factor-kappa B.
