# Supplementary material for: Ma Xing Shi Gan Decoction Attenuates PM2.5 Induced Lung Injury via Inhibiting HMGB1/TLR4/NFκB Signal Pathway in Rat
Source: Front Pharmacol. 2019 Nov 14;10:1361. doi: 10.3389/fphar.2019.01361 (PMC6868102; doi:10.3389/fphar.2019.01361)
Supplement: Supplementary file 1 [file Table_1.docx]

Supplementary Material

## Supplementary Tables

**Supplementary Table 1. Number of animals for different experimental groups**

|  | **Control** | **Intervention** | **PM2.5** | **MXD (g/kg)** | | | **Total** |
| --- | --- | --- | --- | --- | --- | --- | --- |
|  |  |  |  | **4.1** | **8.2** | **16.4** |  |
| **Lung edema** | 8 | 8 | 8 | 8 | 8 | 8 | 48 |
| **Hematoxylin and eosin (H&E) staining** | 6 | 6 | 6 | 6 | 6 | 6 | 36 |
| **Immunohistochemistry (IHC) assay** | (6) | (6) | (6) | (6) | (6) | (6) |  |
| **BALF and serum collection** | 8 | 8 | 8 | 8 | 8 | 8 | 48 |
| **ELISA assay** | (5) | (5) | (5) | (5) | (5) | (5) |  |
| **MDA assay** | (7) | (7) | (7) | (7) | (7) | (7) |  |
| **MPO assay** | (6) | (6) | (6) | (6) | (6) | (6) |  |
| **Western blot** | (3) | (3) | (3) | (3) | (3) | (3) |  |
| **Preparation of medicated serum** | 10 | 10 | - | - | - | - | 20 |
| **Total** | 32 | 32 | 22 | 22 | 22 | 22 | 152 |

The same animals (a total of 36 rats) were used for H&E staining and IHC assay. The same animals (another 48 rats) were used for BALF and serum collection, ELISA assay, MDA assay, MPO assay and Western blot.

**Supplementary Table 2. Elution condition for preliminary UPLC-MS analysis**

| **Time (min)** | **MP A%** | **MP B%** | **Flow rate (mL/min)** |
| --- | --- | --- | --- |
| Initial | 95.0 | 5.0 | 0.300 |
| 2.00 | 95.0 | 5.0 |  |
| 15.00 | 10.0 | 90.0 |  |
| 18.00 | 10.0 | 90.0 |  |
| 19.00 | 95.0 | 5.0 |  |
| 20.00 | 95.0 | 5.0 |  |

For positive electrospray ionization mode, mobile phase solvent A was consisted of aqueous phase of ammonium acetate at 5 mmol/L, and mobile phase solvent B was pure methanol. For negative electrospray ionization mode, mobile phase solvent A was ultrapure water, and mobile phase solvent B was methanol.

**Supplementary Table 3. Elution conditions for ephedrine and pseudoephedrine**

| **Time (min)** | **MP A%** | **MP B%** | **Flow rate (mL/min)** |
| --- | --- | --- | --- |
| Initial | 99.0 | 1.0 | 0.400 |
| 15.00 | 99.0 | 1.0 |  |

Mobile phase solvent A was 0.1% (v/v) formic acid in water, and mobile phase solvent B was acetonitrile.

**Supplementary Table 4. Elution conditions for liquiritin and glycyrrhizic acid**

| **Time (min)** | **MP A%** | **MP B%** | **Flow rate (mL/min)** |
| --- | --- | --- | --- |
| Initial | 98.0 | 2.0 | 0.500 |
| 1.00 | 98.0 | 2.0 |  |
| 2.20 | 70.0 | 30.0 |  |
| 2.50 | 5.0 | 95.0 |  |
| 4.50 | 5.0 | 95.0 |  |
| 4.60 | 98.0 | 2.0 |  |
| 5.00 | 98.0 | 2.0 |  |

Mobile phase solvent A was consisted of aqueous phase of ammonium acetate in concentration 5 mmol/L, and mobile phase solvent B was pure methanol.

**Supplementary Table 5. Elution conditions for amygdalin**

| **Time (min)** | **MP A%** | **MP B%** | **Flow rate (mL/min)** |
| --- | --- | --- | --- |
| Initial | 96.0 | 4.0 | 0.500 |
| 1.00 | 96.0 | 4.0 |  |
| 3.50 | 30.0 | 70.0 |  |
| 4.50 | 30.0 | 70.0 |  |
| 4.60 | 96.0 | 4.0 |  |
| 5.00 | 96.0 | 4.0 |  |

Mobile phase solvent A was 0.1% (v/v) formic acid in water, and mobile phase solvent B was acetonitrile.
